# Remote Sensing Application in Mountainous Environments: A Bibliographic Analysis

**DOI:** 10.3390/ijerph20043538

**Published:** 2023-02-17

**Authors:** Simbarashe Jombo, Mohamed A. M. Abd Elbasit, Anesu D. Gumbo, Nthaduleni S. Nethengwe

**Affiliations:** 1 Department of Physical and Earth Sciences, Sol Plaatje University, Kimberley 8300, South Africa; 2 Risk, Vulnerability and Science Centre, Sol Plaatje University, Kimberley 8300, South Africa; 3 Department of Geography and Environmental Sciences, University of Venda, Thohoyandou 0950, South Africa

**Keywords:** data scarcity, publishing equity, mountain, remote sensing, sustainable development, Africa, bibliometric analysis

## Abstract

Advancement in remote sensing platforms, sensors, and technology has significantly improved the assessment of hard-to-access areas, such as mountains. Despite these improvements, Africa lags in terms of research work published. This is of great concern as the continent needs more research to achieve sustainable development. Therefore, this study applied a bibliometric analysis of the annual production of publications on the application of remote sensing methods in mountainous environments. In total, 3849 original articles between 1973 and 2021 were used, and the results indicate a steady growth in publications from 2004 (*n* = 26) to 2021 (*n* = 504). Considering the source journals, *Remote Sensing* was the top-ranked, with 453 total publications. The University of the Chinese Academy of Sciences was the highest-ranking affiliation, with 217 articles, and China produced the highest number of publications (*n* = 217). Keywords used between 1973 and 1997, such as “Canada”, “alps”, and “GIS”, metamorphosed into “remote sensing” between 1998 and 2021. This metamorphosis indicates a change in the areas of interest and an increase in the application of remote sensing methods. Most studies were conducted in the Global North countries, and a few were published in low-impact journals within the African continent. This study can help researchers and scholars better understand the progress and intellectual structure of the field and future research directions in the application of remote sensing methods in mountainous environments.

## 1. Introduction

Mountain formation has been attributed to plate tectonics, in which pieces of the Earth’s crust smash against each other [1]. The three main types of mountains mainly found in Africa are volcanic, fold, and block [2]. As there is no proper definition of a mountain in the literature, the general understanding is that it is distinctively elevated land compared to the surrounding areas, with steep sides and exposed bedrock [3]. Due to their physical characteristics, mountains provide goods and ecosystem services that humans and animals use [4]. Mountains are one of the most vital ecosystems for the world population because they offer clean water and energy that supports biodiversity [4,5]. The mountain elevation provides for cooler climates and its rugged terrain discourages human intrusion [6]. These characteristics promote the existence of species that are endemic to the area [7].

Many rivers originate from mountains, making them known as “water towers”, supporting more than 2 billion lives worldwide [8]. A good example is the Maluti mountains in Lesotho, which are associated with high precipitation and cloud cover, reduced evaporation, and the provision of fresh potable water to the citizens of the kingdom [9]. Land use and land cover (LULC) and climate changes make mountain ecosystems vulnerable to decay while limiting their provision of ecosystem goods and services [5]. These vulnerabilities have affected the ecological stability of mountains and the livelihoods of communities that access socioeconomic benefits from the mountains [10]. This is especially true in Africa, where most of the population is rural, residing in headwater catchments that are dominated by rugged and mountainous terrain [11]. There exists a beneficial relationship between man and the environment in these areas that can inform management to plan and provide people with equitable and sustainable ecological services [12].

The natural environment offers resources for communities to utilize, and communities, in turn, maintain these environments [13]. Human populations in rural areas depend on freshwater resources of sufficient quantity and quality to sustain their dominant agricultural activities for their livelihoods [14]. The Pungwe River basin in the Zimbabwean part is a good example of a basin that provides livelihood opportunities to inhabitants in the area [11]. The catchment is generally mountainous [15], having the second-highest mountain and the fourth-tallest waterfall in Africa [15]. The area produces enough water resources to cater for the commercial and subsistence farming activities in the area, provides a good environment for fish populations (the most common source of animal protein in rural areas), and promotes ecotourism, which provides jobs for the local communities [16]. Degradation of the mountainous environment that supports this system directly affects the livelihoods of communities that lag behind the successes of sustainable development in Africa [16].

With current projections in climate change trends pointing towards a gloomy future, especially for Africa, it is necessary to fully understand the impact of such change [9,17]. Africa, based on its adaptive capacity, has a limited ability to cope with climate change [2]. The livelihoods of communities in rural Africa are closely related to the availability and use of natural resources, which results in LULC changes that threaten human well-being in the region [18]. Climate change will increase the frequency and magnitude of extreme weather events (e.g., heatwaves, droughts, floods, and hailstorms) [19]. As temperatures increase, some animal species endemic to the cool climates provided by mountainous environments will disappear, and there will be crop damage and failure in these environments [5]. Understanding climate and LULC changes will allow decision-makers to come up with adaptation strategies to combat these alterations [17,20]. However, mountainous regions are data-scarce, a result of their inaccessibility that limits in situ data capturing [21]. However, with modern science, data can be collected and evaluated ex situ to understand changes that a place is undergoing [22]. Remote sensing has become one of the most widely used ex situ data collection and analysis approaches for environmental assessments [10,20,23]. The analysis approach is an alternative source that is quick, easy to use, and intrinsically spatialized [22,24].

Remote sensing is a method of collecting information about objects by analyzing data collected by sensors that are not in contact with the objects of interest [25,26]. The data can be used to map, model, and monitor mountain ecosystem patterns [27]. Moreover, the data can provide comprehensive and cost-effective geospatial information acquired at varying spatial scales, temporal frequencies, and spectral properties [21,22,23]. The application of remote sensing has been successfully used in mountainous environments to classify habitats [28], estimate daily land surface temperature [24], water extraction [29], detect vegetation cover [30], detect fire events [27], evaluate snowpack simulations [31], monitor ecosystem services [5], detect changes in mountain glaciers [32], and mapping mountain forests [33] among many functions. Remote sensing application has advanced where it is used in combination with geographic information systems (GIS) systems in evaluating glacier and permafrost dangers in mountains [33].

Remote sensing methods using optical and radar technology are increasingly crucial for understanding environmental dynamics in mountainous areas [34]. These methods include the use of unmanned aerial vehicles (UAVs), very high spatial and temporal resolution data, and geographical information systems (GIS) data—such as digital elevation models (DEMs)—in modelling, mapping, and monitoring changes in mountainous regions [35,36]. Persistent clouds, frequent and heavy snowfall, and output data accessibility are particularly problematic for optical satellite sensors [37]. These problems can be reduced by using radar sensors that can penetrate clouds and measure mountain deformation rates [38]. With the massive growth in the amount of free and open-access data available, artificial intelligence (AI) and cloud computing are starting to enhance the processing of these new datasets [37]. In mountainous areas, remote sensing has advanced, it has moved beyond simply analyzing images from a single satellite sensor to combining data from several satellite sensors and examining their long-term spatiotemporal properties [39]. Knowledge of the application and use of remote sensing in mountainous environments can help to increase understanding of environmental dynamics [40]. This understanding is useful for decision-makers, natural resource management officers, and other stakeholders in making decisions for conserving and management of resources in mountainous regions.

There has been a rise in research in mountainous areas using passive optical data with high spectral and temporal resolution. By capturing multiple bands and high spectral resolution, the remote sensing data assist in distinguishing features in mountainous regions [41]. Remote sensing advancements have a significant impact on the methods used in monitoring mountainous environments. The use and type of remote sensing data vary from polar to equatorial regions. For example, more observations from the polar regions are seen on the Moderate-Resolution Imaging Spectroradiometer (MODIS) aboard the polar orbit satellite Terra as compared to equatorial regions [42]. Remote sensing methods are convenient in glacial mapping. Satellite imageries, such as IKONOS and Quickbird, have been used to monitor glacial surfaces in three dimensions due to their capability to acquire stereoscopic images, from which elevation data can be extracted [43]. However, their use is constrained at broad spatial scales by their high costs, small swath sizes, and lengthy revisit intervals. Remote sensing is applied to predict future water resources, and glacial hazards and study earth crust movement in mountainous regions [43]. Several indices, such as the Normalized Difference Snow Index (NDSI) and Normalized Difference Vegetation Index (NDVI), are used to separate snow and ice from dark areas such as rocks and monitor vegetation changes [44]. The application of remote sensing methodologies in mountainous environments consists of several processing and analytical techniques. These techniques include image pre-processing, which is important in correcting systematic and non-systematic errors present in remotely sensed images [21,45]. Image post-processing involves the extraction of information from the pre-processed images such as the classification of mountainous environments using either pixel-based or object-based classification methods [21]. Knowledge of the vulnerability of mountain regions to LULC and climate changes and the dependence of communities residing in these regions emphasizes the need for the application of remote sensing strategies in mountain environments [21]. Although mountains are important and fragile, research on mountain environments is still scarce. Existing studies in mountainous environments have been limited and focus on monitoring shifting cultivation [46], measuring, modelling, and monitoring ecosystem services [17], estimation and mapping of soil properties [47], and vulnerability assessments [4]. Understanding possible scenarios of trends in both anthropogenic and natural changes can aid in creating adaptation strategies that are informed by science [32]. This gives a sense of the direction of adaptation that can be followed. Africa is resource-limited and, therefore, requires sound and cost-effective scientific evidence that informs decision-making.

It is against this background that this study applied a bibliometric analysis approach to understand the distribution of relevant literature that applied remote sensing techniques in mountainous environments. This analysis is important because it helps researchers provide an integrated understanding of progress, gaps, directions, and targets for future research studies. Bibliometric analysis popularity is attributed to the advancement, availability, and accessibility of bibliometric software such as VOSviewer 1.6.19 and scientific databases such as Web of Science (WoS) and Scopus [48]. Bibliometric analysis for this study will show the point of view of Africa in terms of research carried out in mountainous areas versus the whole world. The study summarized annual production, source journals, affiliations, collaborations, and country scientific production. Moreover, the study outlined common research topics, co-occurrence networks, and thematic evolution of keywords in publications focusing on the application of remote sensing methods in mountainous environments. The data source, materials, and description of the R statistical software (Version 4.2.2) and packages were used in this study.

## 2. Materials and Methods

### 2.1. Bibliographic Database

**Figure 1:**
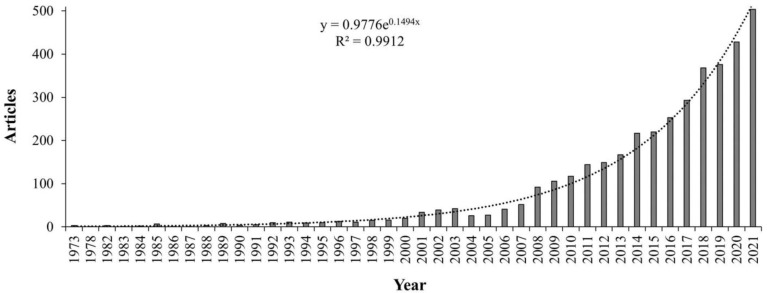
The total number of articles published between January 1973 and December 2021 on the application of remote sensing methods in mountainous environments.

The data used in this study included authors, keywords, citations, source journals, and countries of publications obtained from the Web of Science and Scopus databases. The WoS belongs to Clarivate Analytics and is one of the oldest databases, with more than 1.5 billion references dating back to 1900 [35]. Scopus database has over 17 million researchers profiled, 81 million curated documents, 80,000 institution profiles, and 7000 publishers [36]. For this study, the search for the article was guided by the terms: “mountain”, “mountainous”, and “remote sensing” published between 1 January 1973 and 31 December 2021. A total of 3343 original articles were downloaded from the WoS database and 660 from the Scopus database (Figure 1). The downloaded articles were merged, 154 duplicate articles were removed, and 3849 articles were retained.

### 2.2. R Statistical Application

The bibliometric package in R statistical software analyzed the data generated from the databases. The bibliometrix R-package is written in the R language, which is freely available for generating bibliometric maps using effective statistical algorithms [49]. The data transported into R were translated into a bibliographic data frame and structured for duplication, and the duplicated records were presented as a single document. The bibliometrix package analyzed data by creating a bibliographic coupling, collaboration, co-citation, and co-occurrence network. Spelling errors in articles and associations were checked and corrected before visualizing the author’s names, keywords plus, and keywords.

## 3. Results

### 3.1. Publication Time Series Analysis

From 1973 to 2021, a total of 3849 articles were published focusing on remote sensing and mountain studies. Figure 1 shows how the articles were distributed for the selected period of study. There was minimal production of articles between 1973 and 1989. A steady increase in articles was noticed between 1990 and 2007. An exponential increase was recorded for the period 2008 to 2021. In terms of publishing journals, the top three were *Remote Sensing*, *Remote Sensing of Environment*, and *the International Journal of Remote Sensing* (Table 1). A total of 14 of the top main-source journals were from European countries, 4 were from the USA, 1 from China and 1 from India (Table 1). Table 1 also shows that the top 20 main source journals had 8 journal names including the words “remote sensing”.

### 3.2. Affiliations, Collaborations, Country Scientific Production, and Top-Cited Articles

**Figure 2:**
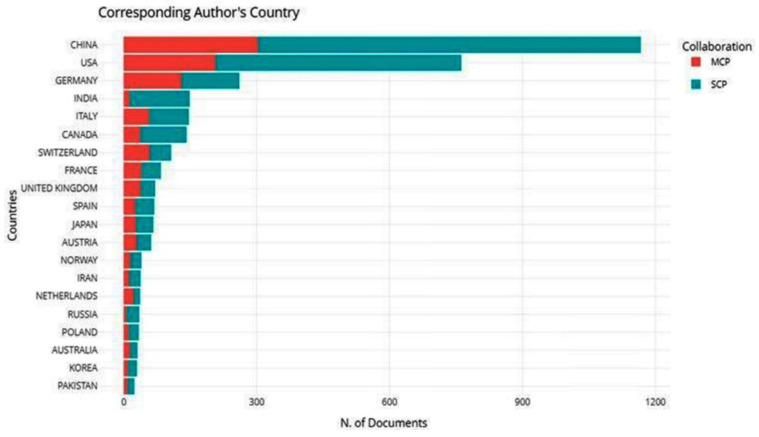
Top 20 countries publishing articles on the application of remote sensing methods in mountainous environments. The country collaborations are represented as inter-country (MCP) and intra-country (SCP) collaboration indices.

**Figure 3:**
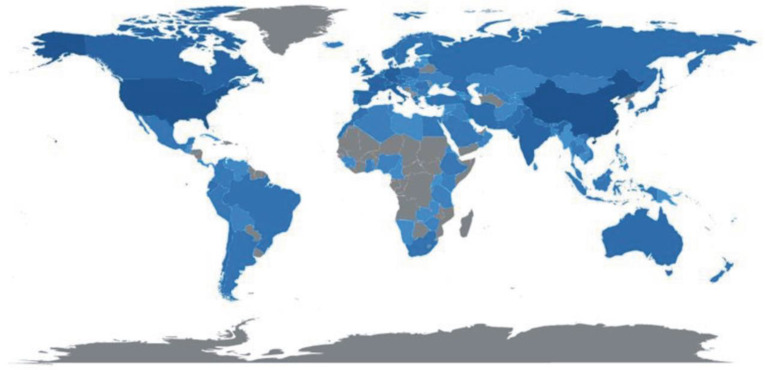
The global distribution of publications in this study.

From these results, 4480 institutions contributed to the analyzed publications on the application of remote sensing techniques in mountainous environments. The University of Chinese Academy of Sciences in China was the highest-ranked affiliation, with 217 articles (Table 2). China and USA were the top-ranked nations, with 9 affiliations that published research on the application of remote sensing methods in mountainous environments (Table 2). Authors affiliated with Chinese institutions produced the highest number of publications on the topic. China had a total of 1167 articles, with an intra-country or single country publication (SCP) collaboration index of 863 and 304 for the inter-country or multiple country publication (MCP) collaboration index, as shown in Figure 2. This indicates that most of the corresponding authors in the published articles on the application of remote sensing methods in mountainous environments were from China. The USA was the second-ranked country, with 762 publications—554 for SCP and 208 for MCP (Figure 2). Most countries had higher SCP compared to MCP values (Figure 2). The global distribution of publications is shown in Figure 3 with most publications produced in China (*n* = 4659) followed by the USA (*n* = 3969) and Germany (*n* = 1088). A few publications were produced in the African continent.

The global distribution of publications is shown in Figure 3 and the darker the color, the more publications have been produced. Most publications were produced in China (*n* = 4659), followed by the USA (*n* = 3969) and Germany (*n* = 1088). A few publications were produced in the African continent, and there was no single publication in Mauritius (Figure 3).

The study revealed that the top five most cited publications focusing on the application of remote sensing methods in mountainous environments were from the *Journal of Glaciology*, *Scientific Bulletin*, *Applied Geography,* and *Remote Sensing of Environment* (Table 3). The top 20 publications cited were written between 2002 and 2019, with a highest total citations (TC) of 587 and citations per year (TCpY) of 65.22 for a publication with Pfeffer as the first author (Table 3). The TC and TCpY for the top 20 cited articles ranged from 87 to 587 and from 7.2 to 73.8, respectively. A total of 14 corresponding authors were affiliated with Chinese institutions; 5 were from the USA, and 1 was from Germany. The *Remote Sensing of Environment* had seven publications, followed by the *Journal of Glaciology* (*n* = 2), and the rest had a single publication in the top 20 cited articles focused on the application of remote sensing in mountainous environments.

### 3.3. Remote Sensing Data Used in the Top 20 Articles Cited

The highest number of studies (*n* = 12) used freely available Landsat satellite images in their studies (Table 4). LiDAR and radar data were used in two studies whilst only a single study used hyperspectral data (Table 4).

### 3.4. Word Cloud, Co-Occurrence Network, and Thematic Evolution

**Figure 4:**
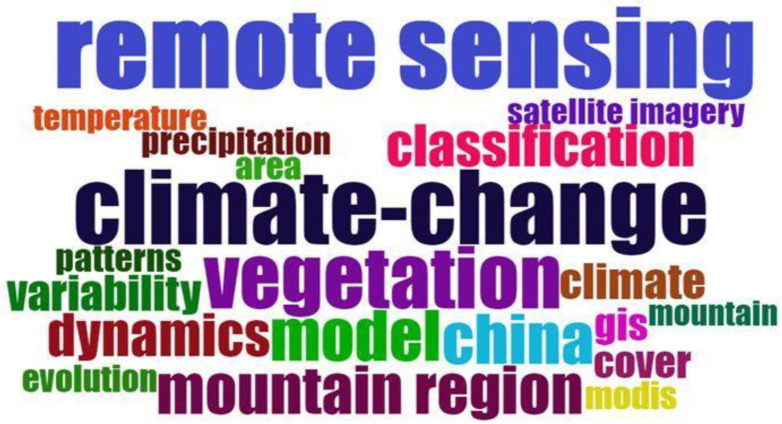
Word cloud showing the top 20 keywords commonly used in studies focused on the application of remote sensing methods in mountainous environments.

The word cloud shown in Figure 4 gives information on the most used keyword in the published articles. The size of the keyword implies the number of occurrences in the publication. Remote sensing was the word most used, with an occurrence of 504 times, followed by climate change (*n* = 304) and vegetation (*n* = 283). Mountain was the 20th-most used word, with an appearance of 115. The closeness of keywords to each other implies their interrelation during the time under investigation.

**Figure 5:**
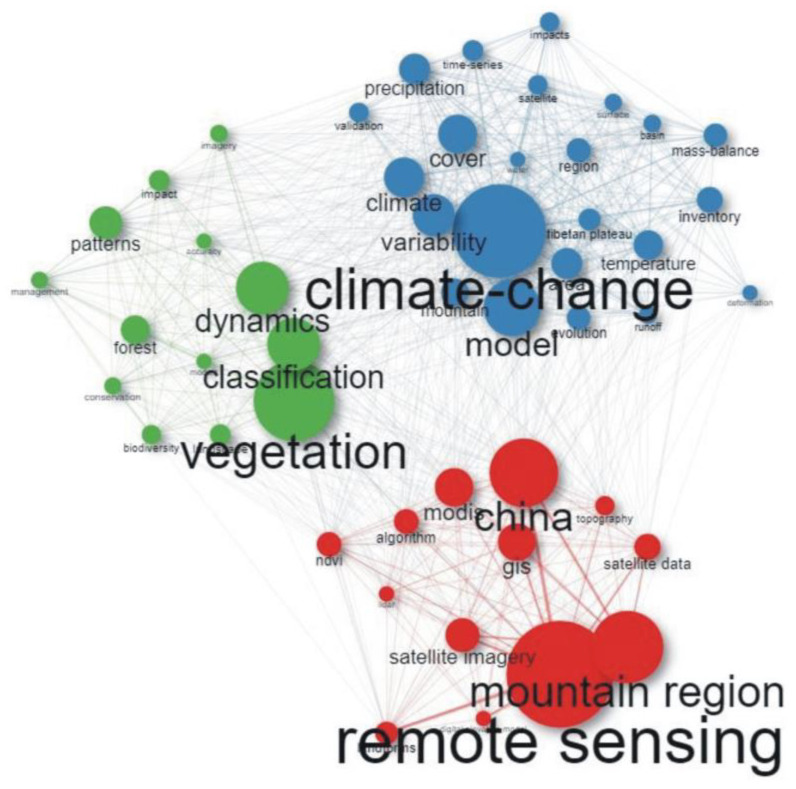
Co-occurrence network for the keywords used in this study. Each node represents a keyword, the size of the node shows the number of occurrences of the keyword, and the thickness of the line shows the degree of connection.

The research theme on the application of remote sensing methods in mountainous environments was categorized into three colored groups. The highest number of keywords were in the blue cluster (*n* = 23), followed by both the red and green clusters (*n* = 13) as illustrated in Figure 5. The blue cluster has keywords including “climate change”, “model”, “variability”, “climate”, and “cover”, while the red cluster had keywords including “remote sensing”, “mountain region”, “China”, “satellite imagery”, and “modis”. The keyword most used was “remote detection”, followed by “climate change”, which were in the red and blue groups, respectively (Figure 5). There was a great connection between “remote sensing”, “China”, and “mountain region” in the red group (Figure 5).

**Figure 6:**
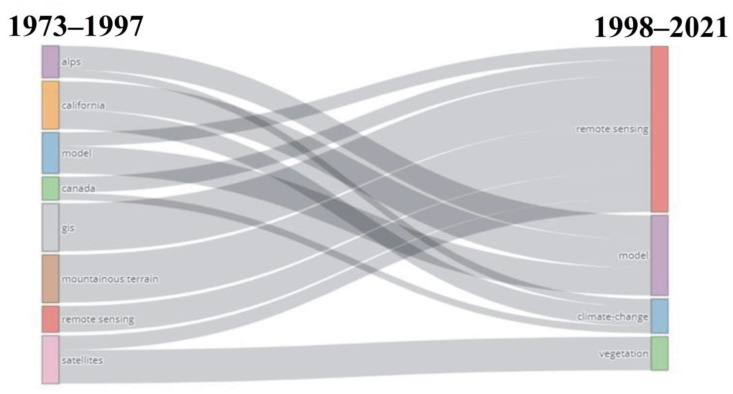
Thematic evolution of keywords from 1973 to 2021 for the publications used in this study.

Thematic evolution of keywords shows that five keywords (“model”, “Canada”, “GIS”, “mountainous terrain”, and “satellites”) metamorphosed between 1973 and 2017 into “remote sensing” between 1998 and 2021 (Figure 6). The keywords “alps”, “California”, and “model” metamorphosed into “model” between 1998 and 2021. The keywords “Canada”, “California”, and “alps” also metamorphosed into “climate change” during the 1998–2021 period. The keyword “satellites” changed to “remote sensing”, and “vegetation” during the 1998–2021 period (Figure 6).

## 4. Discussion

### 4.1. Bibliographic Analysis

The results obtained from a thorough search of the application of remote sensing methods in mountainous environments between 1973 and 2021 showed significant growth in publications. This is a clear indication that more researchers are interested in applying remote sensing methods in mountainous environments. Gathering information about the Earth using remote sensing methods has seen significant evolution since the 1800s [70]. At the time, what appears primitive today was the technology of the time, utilizing pigeons, kites, and hot air balloons to gather information about the earth [71]. Technological advancements in the 20th century saw the invention of airplanes [20]. Concurrently, photography was also developing enabling the capturing of aerial photographs [72]. The need to accurately map how the land surface looks were enhanced by the invention of satellite technology in the 1970s [70]. Since then, the images have been enhanced, and with an increase in technology and understanding systems, satellite imagery has helped scientists understand the environment, detect changes, and predict likely future scenarios [27,45,57]. Mountainous region studies have manipulated the remotely sensed data and have brought about a better understanding of these once hard-to-reach areas [5,10,28]. This evolution in remote sensing from simple tools to highly sophisticated satellite images and, currently, drone-based images producing ultra-high-resolution data [10,35] is part of the reason why the study saw the trend that was followed in Figure 1. In recent times, the use of airborne and spaceborne sensors is replaced by the use of UAVs and small unmanned aerial vehicles (sUAVS) that collect high-quality aerial image data that can help in managing mountainous regions [73]. More articles can now be produced compared to previous decades, when science was still being developed. It is expected that this trajectory will be followed in the future as systems are understood, procedures are refined, and the use of technology is enhanced.

The distribution of research that uses remote sensing in mountainous areas shows that developed nations are the main contributors (Figure 2). China, Europe, and the USA have significant work that utilizes remote sensing work compared to other regions. In Africa, there is a distinct disconnect in the production of this work, as shown in Figure 2. This can be explained by several factors that are inherent to the African situation. Socio-economic and political vulnerabilities have crippled Africa’s progress to contribute to the body of knowledge. During the 1970s, most African countries were battling colonial inequalities, and those who had gained independence were often plunged into civil unrest because of power dynamics [74]. This rendered the continent to contribute late to scientific research. Access is another factor that has resulted in Africa not producing much research on remote sensing applications in mountainous areas [75]. Most technologies are developed and maintained by developed nations such that there are limited native scientists conversant with the procedures and application of remote sensing [76].

Data scarcity has been reported to be a major barrier to environmental assessments in the region [2,9,45]. Remotely sensed data would become the most obvious to use when understanding systems in these data-scarce regions. However, copyrights and ownership of remotely sensed data limit these regions from acquiring quality data to conduct research [77]. This puts Africa in a lagging position as the continent depends on free datasets, which usually have a coarse resolution [78,79]. As images are rendered obsolete in developed nations, they are made available to developing nations for free [80]. Though new to these countries, the information will not have temporal significance, resulting in research based on these datasets not qualifying for publication in reputable journals with a wide readership [81]. Limitations in research publications of this nature in Africa can also be attributed to language barriers [82]. The journals studied are English-based, which limits contributions from French-speaking nations. This is elaborated in Figure 2 without research from the central and west African regions. However, it is different for countries such as South Africa and Zimbabwe because of their well-established tertiary education system [83], the use of English as a language, and government initiatives that promote scientific research [84].

### 4.2. Associations and Production

The top five affiliations where the publications in this study were produced are from China. Chinese institutions published more studies because Chinese researchers use advanced remote sensing applications to monitor mountainous environments and almost two-thirds of the country is high in elevation [23]. This is supported by Li, Pei, Zhao, Xiao, Sang, and Zhang [20], who highlighted that remote sensing methods are being applied by Chinese institutions, including government agencies, research organizations, and universities, in monitoring mountainous environments.

Most publications written by intra-country authors were also in China, with an SCP collaboration index of 863. From the perspective of the spatial distribution of publications in the world, China, the USA, and Germany were the countries with the most publications (Figure 3). This highlights that China has invested so much in science and development; hence, they have the most research studies that focus on the applications of remote sensing methods in mountainous environments. A total of 8 out of 10 countries with high SCP and MCP values are from the Global North, consisting of wealthy and technologically advanced countries. This is in line with Wang, Zhao, and Wang [19], who highlight that most developed papers are from the Global North because they are wealthy and their governments assist them in promoting science and development through research and publication.

The most cited author is Pfeffer et al. [50], with an article published in the *Journal of Glaciology* that has a TC of 587 (Table 3). They were followed by Guo, Liu, Xu, Wu, Shangguan, Yao, Wei, Bao, Yu, Liu, and Jiang [51] who published in the *Scientific Bulletin* journal with a TC of 295 and a TCpY of 73.8 (Table 3). In these studies, remote sensing methods were applied in glacier mountains, and this shows their potential to the global audience, hence high TC and TCpY rates. Other papers applied remote sensing methods in several areas, including urbanization, forestry, land cover changes, glacial lake changes, climate change, and rainfall estimations in mountainous environments [53,55,57,62,63,66]. Weiss and Walsh [21] highlighted that remote sensing applications in mountainous environments continue to be common because of the ongoing and improving utility of imageries in solving real-world problems and providing solutions that can support sustainable mountain management.

### 4.3. Cloud of Words, Co-Occurrence Associations, and Thematic Progress

Remote sensing is the commonly used keyword, followed by “climate change”. The keyword “remote sensing” is close to “satellite imagery”, “temperature”, and “classification” (Figure 4). This shows that studies have been growing over the years, focusing on the application of remote sensing methods using satellite imageries by employing classification techniques. This is in line with Praticò et al. [85], who highlighted that there have been developments in the application of remote sensing methods in the classification of data using satellite imageries in mountainous environments. China was the only country on the word cloud that showed that most studies were conducted in this mountainous nation. Zhao, Bian, and Li [35] highlight that since 2002, the application of remote sensing methods and digital elevation techniques has gradually increased over the years in China for various reasons, including the availability of research funds and technological advances.

The keywords used in the studies are connected, with “remote sensing”, “climate change”, and “vegetation” as the most common words (Figure 5). These keywords are connected because of the increase in remote sensing studies focusing on topics of great interest, including climate change and vegetation studies. This is in agreement with Xu et al. [86], who highlighted an increase in remote sensing research studies focusing on vegetation ecosystem response to changing climate in the 21st centuryThis also explains the metamorphosis of the keywords: “alps”, “California”, “model”, “Canada”, “GIS”, “mountainous terrain”, “remote sensing”, and “satellites” for the period 1973–1997 into “remote sensing”, “model”, “climate change”, and “vegetation” for the 1998–2021 period. Other keywords that were in the co-occurrence network include “classification”, “mountain region”, “gis”, “modis”, and “forest”, which are some of the areas and data sets that are of great concern in recent studies.

### 4.4. Importance of the Study

In recent years, with the development and advancement of satellite imagery, sensors, and techniques, the application of remote sensing methods has been broadly used in the monitoring and management of mountainous environments [4]. The research studies in mountainous environments using remote sensing methods provide knowledge and information that can improve the level of monitoring and formulation of policies that assist in the management and promotion of sustainable mountain management [35]. However, with all these advances, African research in this regard still lags. Several factors affect the ability of the continent to produce internationally recognized papers. The lag in terms of technology and economics renders the research of developing countries “old news” for publishers. However, what is important is not the level of sophistication of methods employed in the African context but rather an understanding of processes and how they impact their livelihoods. If the information on Africa is published in journals with a wide readership, the dissemination of information will increase. This will show gaps in science and promote sustainable development efforts in the region. With literature e.g., [4,9,17,19,45] pointing out that Africa will be greatly impacted by the effects of climate change, it only becomes the next best idea to improve and promote research coming out of the continent.

### 4.5. Limitations

Despite the success of this study in showing the literature that has been published over the years, some limitations were encountered. The study excluded some publications from government agencies, nature conservancies, intergovernmental organizations, and other institutions that have information on the application of remote sensing methods in mountainous environments. Expanding the search across other databases, such as Google Scholar, will help improve the analysis results. The other limitation is that the results were all based on publications authored in the English language. Due to the high volume of publications in China, there might be other studies written in Chinese characters that were not used. This limitation can be addressed by including various languages while searching the research databases.

## 5. Conclusions

This study analyzed global research and publication trends for remote sensing applications in mountainous environments from 1973 to 2021. The study comprised, among others, annual production, source journals, affiliations, collaborations, countries, citations, and keywords. The results indicated steady growth in the number of publications since 2004 and the main source journal was *Remote Sensing*, with a total of 415 publications. Considering the affiliations, the University of Chinese Academy of Sciences was top-ranked, with 217 articles, and China produced the highest number of publications (*n* = 4659). This provides China with a leading position to strengthen other countries on the application of remote sensing in mountainous environments through more collaborations, funding and the provision of technological knowledge. The top-cited article had authors from various countries in the Global North, with a TC of 587 and a TCyP of 65.2. The countries in the Global North can assist other countries in the Global South, especially developing countries in Africa, with resources and funding to develop research using remote sensing methods in mountainous environments. The interconnections and evolution of the keywords over the years hint at the relatedness of remote sensing, climate, and vegetation for future studies. This study offers knowledge of development trends and hotspots that can help in future research that focuses on the application of remote sensing methods in mountainous environments. Research from all countries should strengthen collaborations and exchange of ideas to increase the number of studies focused on topical issues that need remote sensing applications in mountainous environments.

## Figures and Tables

**Table 1:** Top 20 main source journals, country, and the total number of publications.

Rank	Journal Name	Country	Number
1	*Remote Sensing*	Switzerland	415
2	*Remote Sensing of Environment*	USA	222
3	*International Journal of Remote Sensing*	UK	181
4	*Journal of Mountain Science*	China	154
5	*IEEE Transactions on Geoscience and Remote Sensing*	USA	66
6	*Geomorphology*	Netherlands	58
7	*Journal of Glaciology*	UK	50
8	*Mountain Research and Development*	Switzerland	50
9	*IEEE Journal of Selected Topics in Applied Earth Observations and Remote Sensing*	USA	46
10	*Arabian Journal of Geosciences*	Germany	43
11	*ISPRS Journal of Photogrammetry and Remote Sensing*	Netherlands	40
12	*International Journal of Applied Earth Observation and Geoinformation*	Netherlands	39
13	*Canadian Journal of Remote Sensing*	UK	38
14	*Forest Ecology and Management*	Netherlands	37
15	*Cryosphere*	Germany	34
16	*Environmental Earth Sciences*	Germany	34
17	*Hydrological Processes*	UK	33
18	*Forests*	Switzerland	32
19	*Journal of Geophysical Research Atmospheres*	USA	32
20	*Journal of the Indian Society of Remote Sensing*	India	31

**Table 2:** The rank of the top 20 affiliations, country, and the number of articles published.

Rank	Affiliations	Country	Articles
1	University of Chinese Academy of Sciences	China	217
2	Beijing Normal University	China	209
3	The Institute of Mountain Hazards and Environment	China	183
4	Institute of Remote Sensing and Digital Earth	China	175
5	Chinese Academy of Sciences	China	136
6	University of Colorado Boulder	USA	120
7	Institute of Geographic Sciences and Natural Resources	China	109
8	Jet Propulsion Laboratory	USA	94
9	Northwest Institute of Eco-Environment and Resources	China	91
10	University of Maryland	USA	90
11	University of Idaho	USA	89
12	United States Forest Service	USA	88
13	University of Zurich	Switzerland	86
14	Colorado State University	USA	84
15	The University of Arizona	USA	84
16	The University of Oklahoma	USA	83
17	SETI Institute	USA	76
18	University of Marburg	Germany	76
19	Institute of Tibetan Plateau Research	China	75
20	Lanzhou University	China	73

**Table 3:** Top 20 articles cited, corresponding author’s name, year, title, source, total citations (TC), and total citations per year (TCpY)on the application of remote sensing methods in mountainous environments.

Rank	First Author’s Name and Year	Title	Source	TC	TCpY
1	Pfeffer et al. [50]	The Randolph Glacier Inventory: A Globally Complete Inventory of Glaciers	*Journal of Glaciology*	587	65.2
2	Guo et al. [51]	The Second Chinese Glacier Inventory Data Methods and Results	*Journal of Glaciology*		
3	Gong et al. [52]	Stable Classification with Limited Sample Transferring a 30 m Resolution Sample Set Collected in 2015 to Mapping 10 m Resolution Global Land Cover in 2017	*Science Bulletin*	295	73.8
4	Su et al. [53]	Characterizing Landscape Pattern and Ecosystem Service Value Changes for Urbanization Impacts at an Ecoregional Scale	*Applied Geography*	243	22.1
5	Zhu et al. [54]	A Flexible Spatiotemporal Method for Fusing Satellite Images with Different Resolutions	*Remote Sensing of Environment*	235	33.6
6	Xiao et al. [55]	Characterization of Forest Types in NorthEastern China using Multitemporal SPOT4 Vegetation Sensor Data	*Remote Sensing of Environment*	218	10.4
7	Li et al. [56]	Eco-environmental Vulnerability Evaluation in Mountainous Region using Remote Sensing and GIS: A Case Study in the Upper Reaches of Minjiang River China	*Ecological Modelling*	190	11.2
8	Huang et al. [57]	Mapping Major Land Cover Dynamics in Beijing Using All Landsat Images in Google Earth Engine	*Remote Sensing of Environment*	177	29.5
9	Zhang et al. [58]	A 2010 Update of National Land use cover Database of China at 1:100,000 Scale Using Medium Spatial Resolution Satellite Images	*Remote Sensing of Environment*	172	19.1
10	Wulfmeyer et al. [59]	The Convective and Orographically-induced Precipitation Study (COPS): The Scientific Strategy, The Field Phase, and Research Highlights	*Quarterly Journal of the Royal Meteorological Society*	148	12.3
11	Chen et al. [60]	A Mangrove Forest Map of China In 2015 Analysis of Time Series Landsat 78 and Sentinel1A Imagery in Google Earth Engine Cloud Computing Platform	*ISPRS Journal of Photogrammetry and Remote Sensing*	139	23.2
12	Muno et al. [61]	A Catalog of Xray Point Sources from Two Megaseconds of Chandra Observations of the Galactic Center	*Astrophysical Journal Supplement Series*	134	9.8
13	Nie et al. [62]	A Regional-scale Assessment of Himalayan Glacial Lake Changes Using Satellite Observations From 1990 to 2015	*Remote Sensing of Environment*	121	20.2
14	Ma et al. [63]	Response of Hydrological Processes to Landcover and Climate Changes in Kejie Watershed Southwest China	*Hydrological Processes*	114	8.1
15	Li and Sheng [64]	An Automated Scheme for Glacial Lake Dynamics Mapping using Landsat Imagery and Digital Elevation Models: A Case Study in the Himalayas	*International Journal of Remote Sensing*	113	10.3
16	Chen et al. [65]	Forested Landslide Detection Using Lidar Data and the Random Forest Algorithm: A Case Study of the Three Gorges China	*Remote Sensing of Environment*	109	12.1
17	Yin et al. [66]	An Assessment of the Biases of Satellite Rainfall Estimates over the Tibetan Plateau and Correction Methods Based on Topographic Analysis	*Journal of Hydrometeorology*	108	7.2
18	Zhang et al. [67]	Regional Differences of Lake Evolution Across China During 1960s–2015 and its Natural and Anthropogenic Causes	*Remote Sensing of Environment*	107	26.8
19	Jiapaer et al. [68]	Vegetation Dynamics and Responses to Recent Climate Change in Xinjiang using Leaf Area Index as an Indicator	*Ecological Indicators*	100	12.5
20	Yao et al. [69]	Spatiotemporal Pattern of Gross Primary Productivity and Its Covariation with Climate in China Over the Last Thirty Years	*Global Change Biology*	87	17.4

**Table 4:** Overview of the total number of studies, sensor type, and cost of the remote sensing data used in the top 20 articles cited in the application of remote sensing in mountainous environments.

Data Type	Number of Studies	Sensor	Acquisition Cost
Landsat	12	Multispectral	Free
Sentinel	2	Multispectral	Free
MODIS	2	Multispectral	Free
Meteosat Second Generation-8 (MSG-8), LiDAR and radar	1	Multispectral, LiDAR, and Radar	Free and High
SPOT	1	Multispectral	Free
Tropical Rainfall Measuring Mission (TRMM)	1	Radar	Free
LiDAR	1	LiDAR	High
Advanced CCD Imaging Spectrometer (ACIS)	1	Hyperspectral	Free

## Data Availability

Not applicable.
